# A real-world study of polyenyl phosphatidylcholine in the management of patients with metabolic dysfunction-associated fatty liver disease in China clinical practice

**DOI:** 10.3389/fmed.2025.1610083

**Published:** 2025-09-10

**Authors:** Yi Pan, Feng Xue, Shanshan Wang, Bihui Zhong, Yutao Zhan, Qi Wang, Youqing Xu, Huiying Rao, Yuqiang Mi, Yuemin Nan, Xiaoyuan Xu, Branko Popovic, Xiaoqing Li, Bruno Scarpellini, Sabine Tong, Lai Wei

**Affiliations:** ^1^Beijing Tsinghua Changgung Hospital, Tsinghua University, Beijing, China; ^2^School of Clinical Medicine, Tsinghua University, Beijing, China; ^3^Beijing North Medical and Health Economic Research Center, Beijing, China; ^4^Department of Gastroenterology, The First Affiliated Hospital, Sun Yat-sen University, Guangzhou, Guangdong, China; ^5^Department of Gastroenterology, Beijing Tongren Hospital, Capital Medical University, Beijing, China; ^6^Center of Liver Diseases, Beijing Ditan Hospital, Capital Medical University, Beijing, China; ^7^Department of Gastroenterology, Beijing Tiantan Hospital, Capital Medical University, Beijing, China; ^8^Peking University Hepatology Institute, Peking University People's Hospital, Beijing, China; ^9^Clinical School of the Second People's Hospital, Tianjin Medical University, Tianjin, China; ^10^Department of Hepatology, Tianjin Second People's Hospital, Tianjin, China; ^11^Department of Traditional and Western Medical Hepatology, Hebei Medical University Third Hospital, Shijiazhuang, China; ^12^Department of Gastroenterology, Peking University First Hospital, Beijing, China; ^13^Nattermann & Cie. GmbH, Frankfurt am Main, Germany; ^14^Opella, Beijing, China; ^15^Opella, São Paulo, Brazil; ^16^Opella, Neuilly-sur-Seine, France

**Keywords:** China, essential phospholipids, hepatic fibrosis, metabolic dysfunction-associated fatty liver disease, MAFLD, polyenyl phosphatidylcholine, real-world study

## Abstract

**Background/Objectives:**

Metabolic dysfunction-associated fatty liver disease (MAFLD) is highly prevalent in China. Clinical evidence supporting the role of polyenyl phosphatidylcholine (PPC) in delaying liver fibrosis in patients with MAFLD is limited. Hence this study evaluated the effectiveness of PPC and its association with delaying progression of liver fibrosis in patients with MAFLD in China.

**Methods:**

This multicenter, retrospective observational study included patients with type 2 diabetes mellitus or ≥2 metabolic dysregulations. Patients from the MAFLD cohort were divided into two groups to receive either PPC or control (no hepatoprotective treatment). The primary endpoint was the change in baseline fibrosis (FIB)-4 index at 12 and 24 weeks. The secondary endpoint involved comparison of changes in liver enzymes and blood lipid levels.

**Results:**

Among 22,705 patients with MAFLD who were treated with hepatoprotective drugs, 7,093 received PPC. Significant reduction in baseline fibrosis was observed at 24 weeks (PPC: −0.12 ± 0.62 vs. control: 0.11 ± 0.50, *p* = 0.034). Baseline aspartate aminotransferase (AST) levels significantly improved at 12 weeks (PPC: −6.25 ± 15.18 vs. control: −2.41 ± 15.40; *p* = 0.0392). In the PPC group, baseline alanine transaminase (ALT) levels decreased at 12- and 24-weeks compared to those of the control group, but results were not significant. PPC significantly reduced baseline total bilirubin at 12 weeks (*p* = 0.0122) and 24 weeks (*p* = 0.0010), and low-density lipoprotein cholesterol levels at 12 weeks (*p* = 0.0442).

**Conclusion:**

PPC treatment can lower the risk of liver fibrosis and improve liver function and lipid profiles. Further validation is warranted in other ethnic groups in larger cohorts.

## 1 Introduction

Metabolic dysfunction-associated fatty liver disease (MAFLD) is the leading cause of chronic liver disease among adults globally, with an estimated overall prevalence of 32.4% (1, 2). In China, nearly 30.0% of the population is affected by fatty liver disease, which is projected to exceed 314.58 million by 2030 (3–5). MAFLD—a multisystemic disease—encompasses a spectrum of liver-related disorders, progressing from simple steatosis with or without mild inflammation, to metabolic dysfunction-associated steatohepatitis (MASH), with necroinflammation and accelerated fibrosis advancement, eventually leading to cirrhosis and hepatocellular carcinoma (HCC) (1, 6, 7). MAFLD is more prevalent in patients with metabolic comorbidities such as obesity, type 2 diabetes mellitus (T2DM), metabolic syndrome, cardiovascular disease (CVD), and dyslipidemia (1, 6). These conditions may increase the risk of cirrhosis and related complications. Various metabolic elements (insulin resistance, glucotoxicity, and lipotoxicity) along with genetic and other factors contribute to the development and co-occurrence of MAFLD and T2DM, increasing the risk of life-threatening liver complications (8, 9). Previous studies have reported increased likelihood of progression to serious liver problems, such as MASH, advanced fibrosis, cirrhosis, and HCC, in patients with both T2DM and MAFLD (8, 9).

Several studies have reported improved correlation of significant liver fibrosis (≥F2) with MAFLD diagnosis using non-invasive tests (NITs) (9–12).

Various guidelines recommend fibrosis-4 (FIB-4) index as the primary noninvasive test because of its simplicity and cost-effectiveness (13–15). In patients with both MAFLD and T2DM, clinicians should consider screening for clinically significant fibrosis (stages F2–F4) using the FIB-4 index, even if liver enzyme levels are normal (14). The FIB-4 index estimates the risk of hepatic fibrosis based on age, plasma aminotransferase (aspartate transaminase [AST] and alanine transaminase [ALT]) levels, and platelet count (14).

Although liver fibrosis in patients with MAFLD is a severe public health burden, sufficient approved pharmacological therapies are not available. Resmetirom is the only therapy that has recently been conditionally approved by the U.S. FDA for treatment of patients with MASH and moderate-to-advanced hepatic fibrosis (16). Several widely used hepatoprotective drugs for liver injury in China, including silymarin (Silybin), polyenyl phosphatidylcholine (PPC), bicyclol, glycyrrhizic acid preparations (e.g., magnesium isoglycyrrhizinate, diammonium glycyrrhizinate, etc.) might be used in patients with liver biopsy-proven MASH and/or significant fibrosis, or patients with persistently elevated liver enzymes or NITs suggesting a risk of advanced fibrosis (17). PPC is a highly purified active pharmaceutical ingredient extracted from soybean-. PPC influences membrane-dependent cellular functions and demonstrate anti-inflammatory, antioxidant, and antifibrotic effects, thereby improving hepatic regeneration (18). Several studies have reported that PPC could be effective in patients with MAFLD associated with metabolic comorbidities (19–23). Among the treatments investigated for MAFLD, PPC has exhibited potential hepatoprotective effects (20, 24, 25) and is recommended in the Russian (26) and Chinese (27) MAFLD guidelines. PPC plays a role in decreasing elevated liver enzymes (ALT/AST levels), improving abnormalities on ultrasound findings, and reducing blood lipid levels, including levels of total cholesterol, low-density lipoprotein cholesterol (LDL-C), and triglycerides (TG) and liver stiffness and fibrogenesis (22, 24, 25). However, the effectiveness of PPC in improving liver fibrosis in Chinese patients with MAFLD has not been investigated yet.

The aim of this retrospective, observational real-world study was to evaluate the effectiveness of PPC in slowing the course of liver fibrosis by comparing the PPC-treated group with the non-hepatoprotective drug-treated group (control group; received routine comprehensive treatment) in the current clinical settings in China. This study was conducted during the terminology transition period. While we acknowledge “MASLD” as the current updated terminology, the term “MAFLD” has been preferred in this manuscript. This reflects the study’s context, as it was conducted in China, where “MAFLD” remains the preferred English term according to the updated Chinese guideline and position statement by Chinese Society of Hepatology (27, 28).

## 2 Methods

### 2.1 Data source

In this retrospective, observational study, the data collected between January 1, 2020, and December 31, 2022, were extracted from a multicenter database, including Hospital Information System data from 11 tertiary hospitals at 6 cities in China (Beijing, Shanghai, Xi’an, Chongqing, Shenyang, and Wuhan). The principal investigators received standardized and normalized medical data for further analysis.

### 2.2 Study design and population

The present study included adult patients (aged ≥18 years) with MAFLD diagnosed as per the diagnosis criteria of APASL 2020 guideline, defined as the presence of hepatic steatosis with any one of the following three conditions: diabetes mellitus or ≥2 metabolic dysregulations. Metabolic dysregulations were defined as follows: (i) blood pressure (BP) ≥ 130/85 mmHg or the usage of specific drug treatment; (ii) plasma TG level ≥1.70 mmol/L or the usage of specific drug treatment; (iii) plasma high-density lipoprotein cholesterol (HDL-c) level <1.0 mmol/L for men and <1.3 mmol/L for women or the usage of specific drug treatment; (iv) prediabetes (fasting glucose level [FPG] = 5.6–6.9 mmol/L or 2-h post-load glucose level [PLG] = 7.8–11.0 mmol/L or glycated hemoglobin (HbA1c) level = 5.7–6.4%); (v) homeostasis model assessment of insulin resistance (HOMA-IR) score ≥2.5; (vi) plasma high-sensitivity C-reactive protein (hs-CRP) > 2 mg/L. Patients with remarkable missing primary study data, including patient ID, age, gender, and information related to disease diagnosis were excluded from the study.

To assess the effectiveness of PPC on liver fibrosis after 12 weeks±18 days and 24 weeks±36 days of treatment, eligible patients from the MAFLD cohort were divided into two groups: (1) patients receiving comprehensive treatment combined with PPC capsules alone (PPC group) and, (2) those with comprehensive treatment but no hepatoprotective drug treatment (control group). As per the product label in China, the PPC capsules (228 mg) were taken orally three times daily, with two capsules at a time. The maintenance dose was reduced to one capsule three times daily, as per physician’s recommendation. The index date was defined as the date of first prescription of PPC for the PPC group and the first eligible patients encounter/service/visit dates that met the inclusion and exclusion criteria for the control group. In both groups, patients with cirrhosis, viral hepatitis, HCC, and other extrahepatic malignancies; patients with significant missing information related to the use of PPC (in PPC group); and patients with no required follow-up period (i.e., 12 weeks±18 days and 24 weeks±36 days after index date) were excluded from the study. The patient’s baseline period was defined from the study start date to the index date.

This study was compliant with the principles of the Declaration of Helsinki and was also approved by the Clinical Research Ethics Committee of Beijing Tsinghua Changgung Hospital (No. 22534-0-01).

### 2.3 Study objectives

The primary objective of the study was to analyze the effectiveness of PPC on liver fibrosis in patient with MAFLD, and the secondary objectives were to describe the clinical features of adult patients with MAFLD treated with PPC in China and evaluate the effectiveness of PPC in improving liver function (liver enzymes levels) among patients with MAFLD. Exploratory objective was to explore the advantages of PPC in improving blood lipids in patients with MAFLD and hyperlipidemia.

### 2.4 Study measures

Demographic information captured on the index date such as age, gender, baseline characteristics (liver enzymes levels, blood levels of lipid, FPG, HbA1c), disease-related information (patient’s extrahepatic disease and liver disease spectrum) and prescribed medications along with PPC were recorded for the MAFLD cohort.

To assess the effectiveness of PPC, the primary endpoint was change in FIB-4 index from baseline to 12 weeks±18 days weeks and 24 weeks±36 days between the PPC and non-hepatoprotective control groups. The secondary endpoint involved comparison of the changes in liver function indicators, such as AST and ALT levels, total bilirubin, from baseline to 12 weeks±18 days and 24 weeks±36 days of treatment between the two groups. Additionally, among patients with MAFLD and hyperlipidemia, the between-group difference for changes in blood lipid levels was compared.

### 2.5 Fibrosis assessment

The FIB-4 score was computed using the existing equation, which included age, AST and ALT levels, and platelets counts: (FIB-4 score = age [years] × AST [U/L]) / ([platelets (10^9^/L)] × (ALT [U/L])^1/2^).

A value of <1.3 (F0–F1) was considered low risk and ruled out advanced fibrosis, whereas a value of 1.3–2.67 (F2) was considered intermediate risk and warranted further assessment via liver stiffness measurement using elastography or other methods. A value of >2.67 was considered high risk of advanced fibrosis (F3–F4) and increased risk of adverse liver outcomes (13, 14).

### 2.6 Statistical analysis

All statistical analyses were performed using the SAS software (Version 9.4; SAS Institute Inc., Cary, NC, USA), with a significance level of *p* = 0.05 and two-sided tests. Continuous variables were expressed as mean±standard deviation (SD) for skewed distribution, the values were presented as median and interquartile range (IQR). Categorical variables were presented as proportion (%) of the study population per category.

For effectiveness analysis, changes in the baseline FIB-4 index at 12 weeks±18 days and 24 weeks±36 days between the PPC and control groups were evaluated using both analysis of covariance (ANCOVA) and propensity-score matching (PSM) methods. We used the 1:1 PSM method to match an equal number of PPC-treated patients with the control based on baseline characteristics, including age, sex, and comorbidities (T2DM, hyperlipidemia, hypertension, CVD, when applicable). Subsequently, ANCOVA was performed to compare the inter-groupchanges in liver enzymes/blood lipid indicators between the PPC and control groups, with the baseline indicators and the period in days between baseline examination and follow-up examination as covariates. This method was used to mitigate selection bias and the influence of covariates on the comparisons.

The change in liver function indicators (ALT, AST, total bilirubin levels) and hyperlipidemia (LDL-C levels) from baseline to those at 12 weeks±18 days and 24 weeks±36 days in the PPC group compared to those in the control group were assessed using both ANCOVA and PSM methods, which was similar to the approach used for the primary endpoint analysis. ANCOVA analyses were performed for each liver function indicator and hyperlipidemia, with the corresponding baseline values used as covariates. The duration in days between the baseline examination and follow-up examination was also included as a covariate.

The representativeness and generalizability of this study may be limited to a specific profile of MAFLD patients due to the requirement of specific laboratory information and the use of real-world data collected in routine healthcare setting. Thus, a sensitivity analysis was performed to compare the baseline characteristics of the excluded population with those of the study population.

## 3 Results

### 3.1 Characteristics of PPC-treated patients with MAFLD

#### 3.1.1 Demographics and laboratory information of PPC-treated patients with MAFLD

A total of 82,908 patients with MAFLD were identified from the database, of which 22,705 (27.4%) patients were treated with hepatoprotective drugs; 7,093 (31.2%) patients were treated with PPC capsules, which is the most commonly used hepatoprotective drug in a real-world setting. Of those, 68.3% of patients PPC-treated were men, and the mean (±SD) age of the patients was 50.40 (±15.61) years. Men were observed to be younger than women in the PPC group (age group: 18–49 years; 59.4% vs. 30.4% for men and women, respectively). Nearly, half of the patients treated with PPC exhibited abnormal AST levels, and 65.6% had abnormal ALT levels. Furthermore, based on the FIB-4- index based fibrosis risk stratification, 47.6% were classified as intermediate and high risk (**Table** 1).

**Table 1:** Demographics and laboratory data of PPC-treated patients with MAFLD.

Characteristics	PPC group (*N* = 7,093)	
Gender	Male (*n* = 4,841)	Female (*n* = 2,252)
Age (years), median (IQR)	57 (47–65)	46 (35–57)
Liver function index		
AST (U/L), median (IQR)	39.00 (25.60–64.0)	
AST > 40, *n* (%)	2,196 (48.2)	
ALT (U/L), median (IQR)	57.40 (30.60–97.7)	
ALT >40, *n* (%)	3,037 (65.6)	
Total bilirubin (μmol/L), median (IQR)	13.70 (10.30–19.2)	
ALP (U/L), median (IQR)	84.00 (68.00–107.0)	
GGT (U/L), median (IQR)	72.06 (42.0–129.8)	
Blood glucose		
FPG (mmol/L), median (IQR)	6.13 (5.32–8.1)	
FPG ≥ 5.6, *n* (%)	944/1478 (63.9)	
HbA1c (%), median (IQR)	6.40 (5.80–7.7)	
HbA1c ≥ 5.7, *n* (%)	1493/1875 (79.6)	
Blood lipid		
HDL-C (mmol/L), median (IQR)	1.02 (0.88–1.2)	
LDL-C (mmol/L), median (IQR)	3.00 (2.36–3.6)	
LDL-C ≥ 3.4, *n* (%)	1409/4142 (34.0)	
TC (mmol/L), median (IQR)	4.78 (4.01–5.6)	
TC ≥ 5.2, *n* (%)	1535/4227 (36.3)	
TG (mmol/L), median (IQR)	2.13 (1.54–3.1)	
TG ≥ 1.7, *n* (%)	2967/4228 (70.9)	
Noninvasive fibrosis markers		
FIB-4 score, *n* (%)		
Low risk (<1.30)	1,882 (52.4)	
Intermediate risk (1.3–2.67)	1,034 (28.8)	
High risk (>2.67)	678 (18.9)	

ALP, alkaline phosphatase; ALT, alanine transaminase; AST, aspartate transaminase; FIB-4, Fibrosis-4; FPG, fasting plasma glucose; GGT, γ-glutamyl transpeptidase; HbA1c, glycated hemoglobin A1c; HDL-C, high-density lipoprotein cholesterol; IQR, interquartile range; LDL-C, low-density lipoprotein cholesterol; PPC, polyenyl phosphatidylcholine; TC, total cholesterol; TG, triacylglycerol.

#### 3.1.2 Metabolic comorbidities and extrahepatic disease in PPC-treated patients with MAFLD

T2DM was the most prevalent comorbidity among PPC-treated patients with MAFLD, affecting over 48.5% of PPC-treated group. CVD was another significant comorbid condition, occurring in almost 41.9% of the participants. Additionally, hyperlipidemia was present in 40.3% of the patients, and hypertension in 38.6% (Table 2).

**Table 2:** Extrahepatic disease in patients with MAFLD treated with PPC.

Extrahepatic disease	PPC group (*N* = 7,093)
T2DM, *n* (%)	3,442 (48.5)
CVD	2,970 (41.9)
Ischemic heart disease	1,061 (15)
Heart failure	478 (6.7)
Stroke	323 (4.6)
Hyperlipidemia	2,857 (40.3)
Hypertension	2,741 (38.6)
Extrahepatic malignancies	441 (6.2)
Osteoporosis	425 (6)
CKD	411 (5.8)
Cholelithiasis	386 (5.4)
Thyroid dysfunction	203 (2.9)
Obstructive sleep apnea	63 (0.9)
Cognitive disorders	47 (0.7)
Polycystic ovarian syndrome	38 (0.5)
Psoriasis	3 (0.04)

CVD, cardiovascular disease; CKD, chronic kidney disease; T2DM, type 2 diabetes mellitus.

#### 3.1.3 Commonly visited departments by PPC-treated patients with MAFLD

Our data indicated that PPC treated patients with MAFLD predominantly visited the specialized departments of endocrinology (20.7%), and hepatology and/or gastroenterology (12.2%). Notably, only a small proportion (2.6%) of patients visited the infectious diseases department (Table 3).

**Table 3:** Departments visited by patients with MAFLD treated with PPC.

Extrahepatic disease	PPC group (*N* = 7,093)
Endocrinology, *n* (%)	1,466 (20.7)
Hepatology/Gastroenterology	862 (12.2)
Cardiology	688 (9.7)
Neurology	309 (4.4)
General Surgery	296 (4.2)
Nephrology	270 (3.8)
Internal medicine	233 (3.3)
Rheumatology and immunology	231 (3.3)
Respiratory medicine	230 (3.2)
Infectious diseases	183 (2.6)
Emergency	166 (2.3)
Traditional Chinese medicine	166 (2.3)
General medicine	124 (1.8)
Geriatrics	84 (1.2)
Others	1785 (25.1)

#### 3.1.4 Other common drugs combined with PPC for patients with MAFLD

Bicyclol was the primary hepatoprotective drug that was used along with PPC capsules and was prescribed for 29.3% of patients undergoing treatment with PPC and other hepatoprotective drugs. Diammonium glycyrrhizinate was administered to 14.4% of patients and compound glycyrrhizin to 13.4% of patients (Table 4). The drugs used most frequently in combination with PPC for treating diabetes, high cholesterol, and hypertension were metformin (25.5%), atorvastatin (33.9%), and amlodipine (8.9%), respectively (Table 4).

**Table 4:** Administration of commonly prescribed drugs used in combination with PPC.

Drugs	PPC combined with other therapeutic drugs for comorbidities or hepatoprotection
Hepatoprotective drugs, *n* (%)	*N* = 2,936
Bicyclol	860 (29.3)
Diammonium glycyrrhizinate	424 (14.4)
Compound glycyrrhizin	392 (13.4)
Ursodesoxycholic acid	373 (12.7)
Glutathione	346 (11.8)
Antidiabetic drugs	*N* = 1899
Metformin	485 (25.5)
Acarbose	119 (6.3)
Linagliptin	97 (5.1)
Metformin and acarbose	83 (4.4)
Dapagliflozin	78 (4.1)
Lipid-lowing drugs	*N* = 2,108
Atorvastatin	738 (33.9)
Rosuvastatin	429 (19.7)
Fenofibrate	311 (14.3)
Pitavastatin	191 (8.8)
Ezetimibe	168 (7.7)
Antihypertensive drugs	*N* = 1,535
Amlodipine	137 (8.9)
Nifedipine	135 (8.8)
Metoprolol	126 (8.2)
Valsartan and amlodipine (I)	118 (7.7)
Irbesartan	71 (4.6)

### 3.2 Effects of PPC on liver function over time

#### 3.2.1 Effects of PPC on FIB-4 index of patients with MAFLD

**Figure 1:**
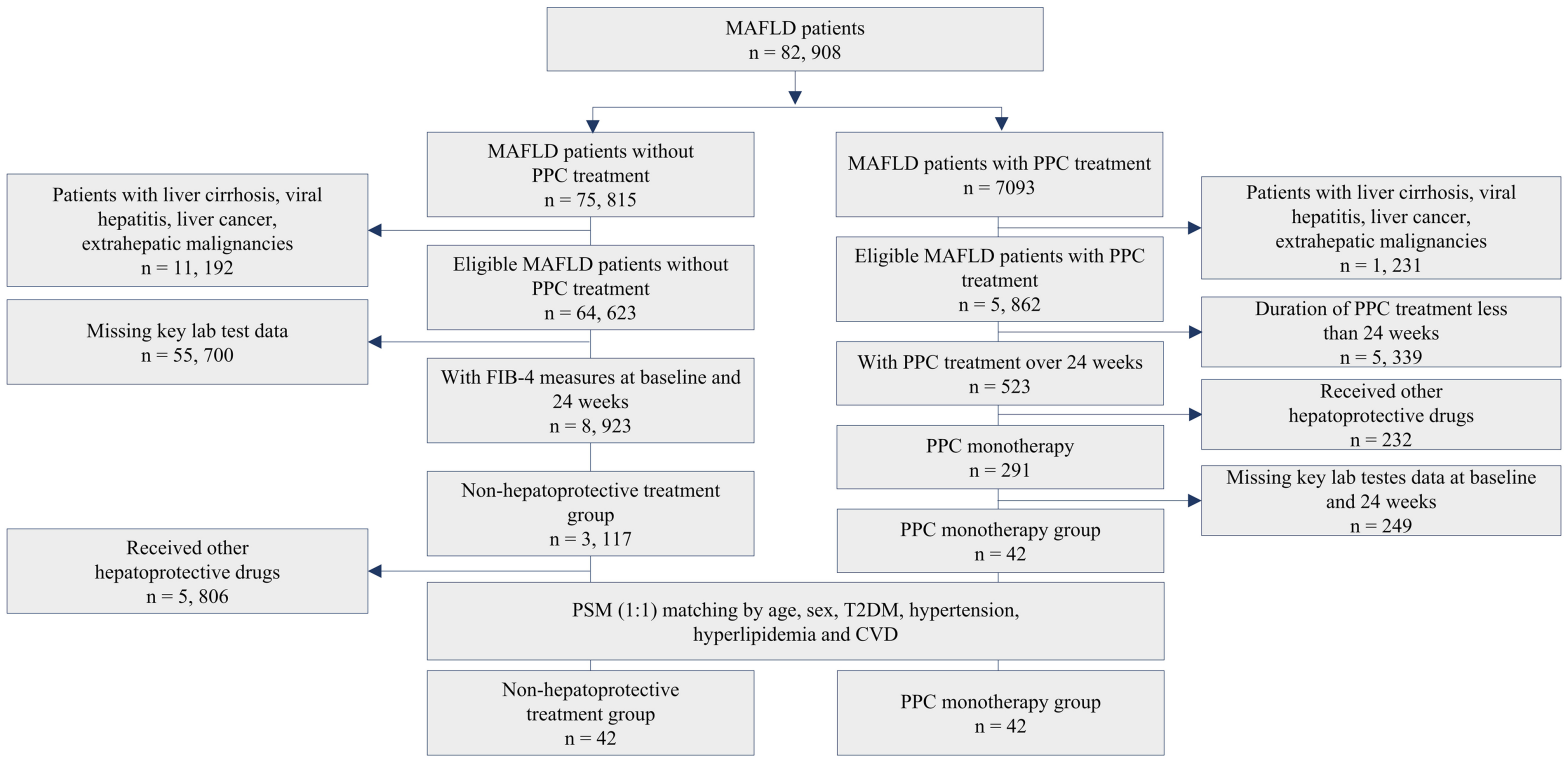
Flowchart of patient selection.

Of the 7,093 patients who received PPC, 291 patients treated with monotherapy for a minimum duration of 24 weeks and did not present with liver cirrhosis, were included in the effectiveness analysis; FIB-4 index data were available for 42 patients. Using PSM, 42 well-matched patients with MAFLD who did not receive hepatoprotective therapy were selected for the control group (Figure 1).

The baseline characteristics of the two groups before and after PSM, including demographics, are summarized in Supplementary Table S1.

**Figure 2:**
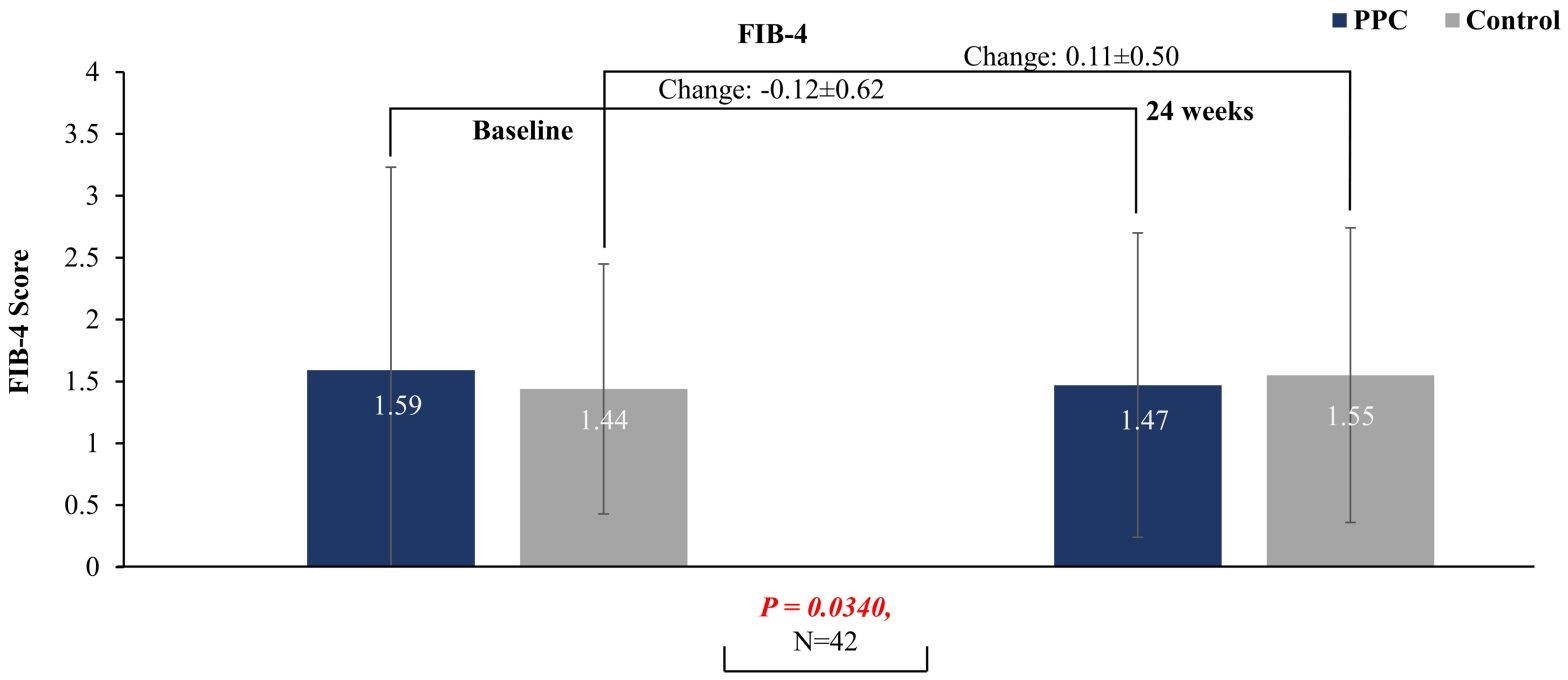
FIB-4 score at baseline and at 24 weeks in PPC and control groups. Data are presented as mean with error bars indicating standard deviation of mean value.

After 24 weeks of treatment with PPC monotherapy, a significant decrease in baseline FIB-4 index was observed to that for the control group (PPC: −0.12 ± 0.62 vs. control: 0.11 ± 0.50; *p* = 0.034), suggesting a beneficial effect of PPC for liver fibrosis. However, a significant difference was not observed in baseline FIB-4 index after at 12 weeks between the two groups (*p* = 0.7381) (Figure 2).

#### 3.2.2 Effects of PPC on liver enzymes of patients with MAFLD

**Figure 3:**
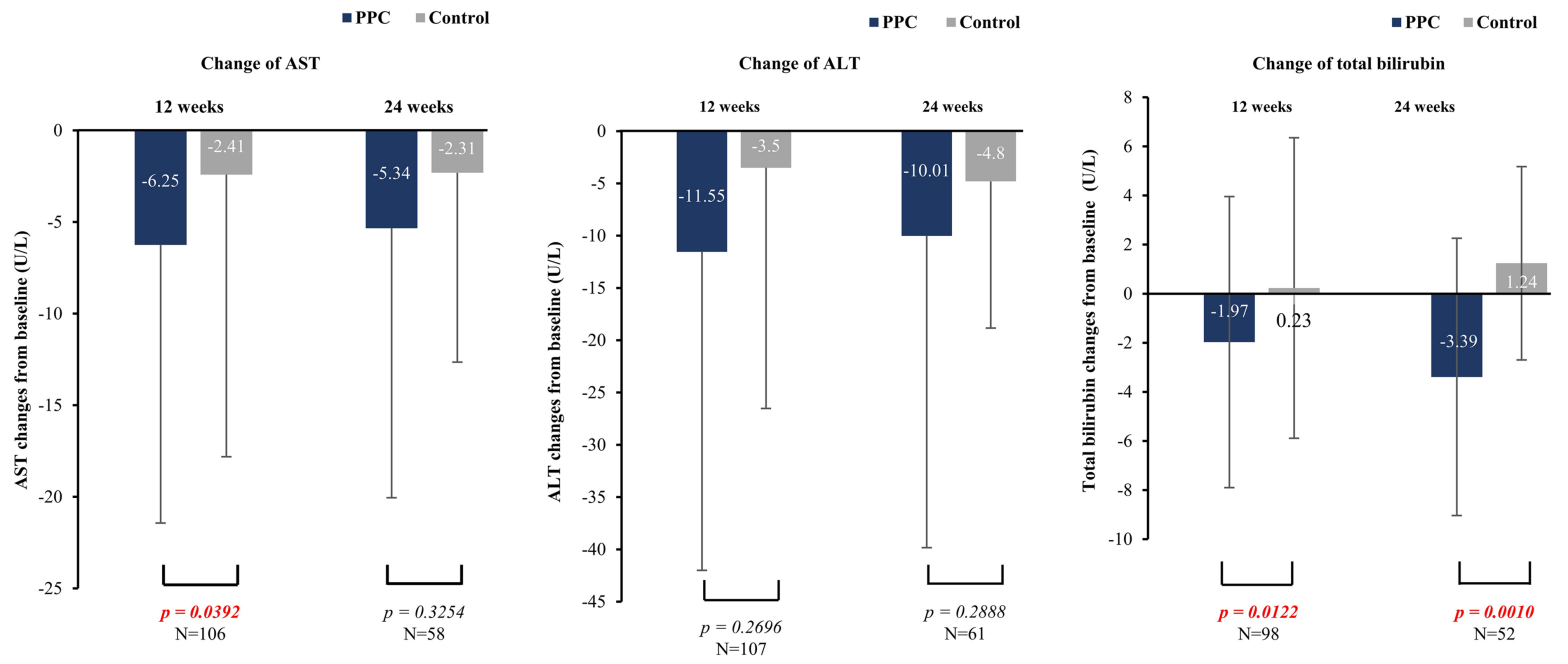
Comparison of changes in liver enzyme levels between PPC and control groups. Data are presented as mean with error bars indicating standard deviation of mean value.

The improvements in baseline AST level at 12 weeks were significantly higher in the PPC group than in the control group (−6.25 ± 15.18 for PPC vs. −2.41 ± 15.40 for control, *p* = 0.0392) (Figure 3). The PPC group showed a decreasing trend in baseline ALT levels at 12- and 24-weeks vs. control group, although this difference was not statistically significant (Figure 3). Significant reduction in baseline total bilirubin levels at both 12 and 24 weeks were observed in the PPC group (12 weeks: −1.97 ± 5.93 for PPC vs. 0.23 ± 6.12 for control, *p* = 0.0122; 24 weeks: −3.39 ± 5.65 for PPC vs. 1.24 ± 3.94 for control, *p* = 0.0010) compared to that in the control group (Figure 3). The baseline characteristics of the two groups before and after PSM, including demographics, are summarized in Supplementary Tables S2–S5.

### 3.3 Effects of PPC on LDL-C of hyperlipidemia sub-group patients with MAFLD

**Figure 4:**
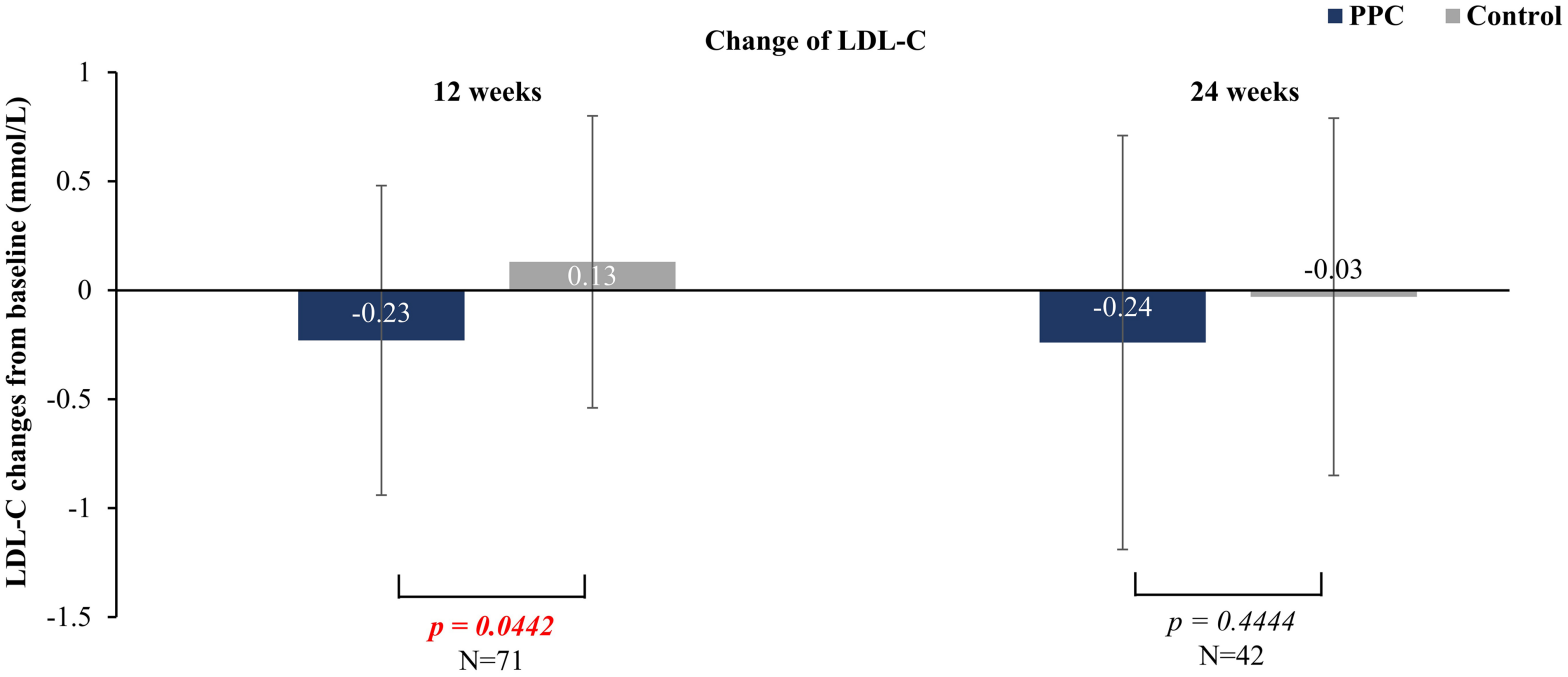
Comparison of changes in LDL-C levels between PPC and control groups. Data are presented as mean with error bars indicating standard deviation of mean value.

In sub-group patients with MALFD and hyperlipidemia, during the 12-week treatment period, the PPC monotherapy group exhibited a significant reduction in LDL-C levels (−0.23 ± 0.71 for PPC vs. 0.13 ± 0.67 for control, *p* = 0.0442) compared to that in the control group (Figure 4) which was not statistically significant at 24 weeks.

### 3.4 Sensitivity analysis

As with other non-randomized studies, our findings may be sensitive to potential selection biases. To address this, we performed a sensitivity analysis to determine if patients included in the effectiveness study differed from all patients receiving PPC monotherapy. The results showed no significant differences in baseline characteristics between the two groups of patients (Supplementary Tables S6, S7).

## 4 Discussion

In this study, we conducted a comprehensive analysis of laboratory parameters, extrahepatic comorbidities, department-visit patterns, liver function tests, and treatment approaches for PPC-treated patients with MAFLD. Additionally, we evaluated the effectiveness of PPC on the liver fibrosis risk indicator FIB-4, biochemical indices of liver function (ALT, AST, total bilirubin), and blood lipid (LDL-C) levels.

In the present study, we observed a notably high prevalence of CVD (41.9%), T2DM (48.5%), hypertension (38.6%), and hyperlipidemia (40.3%) among MAFLD patients treated with PPC. These results are similar to those of previous studies, highlighting that overweight/obesity, hypertension, and hypercholesterolemia are the most prevalent comorbidities associated with MAFLD (21). Moreover, a study from China reported that up to 7.21% of residents had both T2DM and MAFLD, accentuating substantial morbidity and comorbidity of these chronic metabolic conditions (29). This is the first study to analyze departmental visit patterns of patients with MAFLD treated with PPC; we observed that 20.7, 9.7, and 12.2% of the patients underwent treatment in the department of endocrinology, cardiology, and hepatology and/or gastroenterology, respectively. This pattern highlights the complex and multifactorial nature of MAFLD and the importance of promoting a multidisciplinary treatment model for its management.

In our study, 47.6% of patients were classified as having intermediate-to-high risk of clinically significant liver fibrosis (≥F2) as per the FIB-4 index, 48.2 and 65.6% patients reported elevated plasma aminotransferase levels (AST > 40 U/L and/or ALT >40 U/L, respectively). This suggests that PPC is commonly used in MAFLD patients with elevated level of liver enzymes or with the risk of significant fibrosis as per non-invasive tests in real-world clinical settings, which aligned with the 2024 Chinese MAFLD guideline (17). Furthermore, promoting the FIB-4 index as the preferred test for screening and stratification of patients with MAFLD, despite normal liver enzyme levels, is crucial in liver disease-related departments across all tiers of hospitals in China, particularly in primary hospitals and non-liver speciality departments, such as endocrinology and cardiology.

In this study, patients with MAFLD appropriately received hypoglycemic, anti-hypertensive, and hypolipidemic treatments; however, <30% of patients, received hepatoprotective therapy. Currently, in China, hepatoprotective drugs are extensively and safely employed in managing liver injury in patients with chronic liver diseases (27). Nevertheless, the therapeutic impact of these drugs on steatohepatitis and fibrosis in MAFLD requires further confirmation (27).

This study has demonstrated that the FIB-4 index was significantly reduced in the PPC-treated group compared to that in the control group (non-hepatoprotective treated) after 24 weeks of treatment. The PPC group exhibited a decreasing trend of baseline ALT/AST levels at 12- and 24-weeks compared to that in the control group. The findings from this study also demonstrated that PPC treatment for 24 weeks not only decreased the FIB-4 index value but also reduced total bilirubin levels. Similarly, PPC treatment for 12 weeks reduced AST and total bilirubin levels in patients with MAFLD. Moreover, in patients with hyperlipidemia, PPC treatment for 12- and 24-weeks reduced LDL-C levels, indicating its positive effect on ameliorating fibrosis in these patients. Our results align with the findings from other randomized controlled trials that demonstrated delayed progression of hepatic fibrosis upon additional treatment with PPC for 6 months in patients with MASH and diabetes, adequately controlled by sibutramine and metformin (30, 31). In another randomized, active-controlled trial, it was observed that the combination treatment of PPC and pioglitazone for 6 months reduced liver fibrosis more effectively than treatment with pioglitazone alone (32).

To our understanding, this is the first study to directly evaluate the impact of PPC on fibrosis in patients with MAFLD in a real-world setting. In 2020, an observational, multicenter, prospective study was conducted, involving 2,843 newly diagnosed patients with MAFLD and at least one of the four comorbidities (overweight/obesity, hypertension, T2DM, and hypercholesterolemia). The study reported a significant improvement in liver enzymes (AST, ALT, gamma-glutamyl transpeptidase) levels and serum lipid profile (triglyceride, and total cholesterol) upon additional treatment with PPC for 12 and 24 weeks alongside standard of care; however, the efficacy of PPC on fibrosis was not assessed (25).

Previous studies have reported that PPC may hinder collagen accumulation induced by transforming growth factor-β1 in cultured rat hepatic stellate cells and enhance collagen breakdown in cultured lipocytes (33–35). The conversion of lipocytes into transitional cells is a crucial factor in the development of liver fibrosis (34). Furthermore, mitochondrial dysfunction could contribute to liver fibrosis progression (35, 36). PPC, as a membrane-repairing and stabilizing agent, may potentially prevent liver fibrosis by preserving mitochondrial membrane integrity (18, 37). However, this hypothesis requires validation. Additionally, PPC improves AST and LDL-C levels, which may indirectly reduce the progression of liver fibrosis (38, 39). As a hepatoprotective agent, PPC could be administered orally for an extended period, with ongoing evaluation of its treatment effectiveness, as recommended (27).

However, the study also had some limitations. First, the diagnosis of MAFLD in our study was not confirmed through chart review, and the severity of MAFLD could not be established due to the absence of biopsy data or other indexes of fibrosis, such as elastography values. Second, our data was derived from the Health Information System (HIS) and does not include waist circumference measurements or records of overweight/obesity. Thus, we might have underestimated the number of patients with MAFLD in our study since individuals with fatty liver disease who also have overweight/obesity can be classified as having MAFLD. In addition, the clinical characteristics of MAFLD patients when combined with obesityare not well defined. Third, due to the observational nature of the study, inherent patient selection bias is possible. We have matched most of potential confounders including age, sex, T2DM, hypertension, hyperlipidemia and CVD according to previous reports (40) to minimize imbalances, but the hidden effects of unmeasured variables may still have biased the results. Fourth, the sample size was limited due to the nature of the real-world database limiting the availability of specific laboratory information at designated follow-up timepoints, but also because PPC capsules are available over-the-counter in China, and follow-up information is not captured in our hospital database. Thus, the representativeness and generalizability of the findings could be restricted. However, the final sample size ensures that the primary inferential goal of the study is achieved from a statistical standpoint. Lastly, as our data were sourced from Chinese patients, further validation is warranted in other ethnic groups.

## 5 Conclusion

The findings of this study suggest that patients with MAFLD represent a population burdened with high rate of comorbidities, primarily CVD, T2DM, and hypertension. Notably, 47.6% of patients treated with PPC were classified as having medium-to-high-risk of fibrosis according to the FIB-4 index. Currently, significant gaps exist in the management of patients with MAFLD in China. This study elucidates the beneficial effects of PPC capsule on fibrosis risk reduction in patients with MAFLD. The study also reveals that PPC capsule treatment can reduce liver enzymes levels while improving lipid disorders. PPC may serve as a viable treatment option for patients with MAFLD along with significant liver inflammation (elevated ALT/AST level) and those at risk of significant fibrosis, particularly those with T2DM and/or cardiovascular metabolic risk factors.

## Data Availability

The original contributions presented in the study are included in the article/Supplementary material, further inquiries can be directed to the corresponding author.
