# Supplementary material for: A real-world study of polyenyl phosphatidylcholine in the management of patients with metabolic dysfunction-associated fatty liver disease in China clinical practice
Source: Front Med (Lausanne). 2025 Sep 10;12:1610083. doi: 10.3389/fmed.2025.1610083 (PMC12457410; doi:10.3389/fmed.2025.1610083)
Supplement: Supplementary file 1 [file Table_1.DOCX]

Supplementary Material

Table S1: Baseline characteristics before and after propensity score matching (FIB-4 24 weeks)

| **Baseline**  **variables** | **Before PSM** | | |  | **After PSM** | | |
| --- | --- | --- | --- | --- | --- | --- | --- |
|  | **PPC group**  **(n=42)** | **Control group**  **(n=3,117)** | **p-value** |  | **PPC group**  **(n=42)** | **Control group**  **(n=42)** | **p-value** |
| Age (years), mean±SD | 57.98±17.11 | 55.83±14.91 | 0.4242 |  | 57.98±17.11 | 57.00±16.79 | 0.7925 |
| Sex, Male n (%) | 25(59.52) | 1905(61.12) | 0.8740 |  | 25(59.52) | 25(59.52) | 1.0000 |
| T2DM, n (%) | 26(61.9) | 1669(53.55) | 0.3502 |  | 26(61.90) | 27(64.29) | 1.0000 |
| Hypertension,  n (%) | 23(54.76) | 1772(56.85) | 0.8756 |  | 23(54.76) | 23(54.76) | 1.0000 |
| Hyperlipidemia, n (%) | 33(78.57) | 2722(87.33) | 0.1014 |  | 33(78.57) | 33(78.57) | 1.0000 |
| CVD, n (%) | 35(83.33) | 2384(76.48) | 0.3617 |  | 35(83.33) | 35(83.33) | 1.0000 |

FIB-4, fibrosis-4, age ([yr] x AST [U/L]) / ((PLT [10(9)/L]) x (ALT [U/L])(1/2)); PPC, polyenyl phosphatidylcholine; PSM, propensity-score matching; T2DM, type 2 diabetes mellitus; CVD, cardiovascular disease.

Table S2: Baseline characteristics before and after propensity score matching (AST 12 weeks)

| **Baseline variables** | **Before PSM** | | | **After PSM** | | |
| --- | --- | --- | --- | --- | --- | --- |
|  | **PPC group**  **(n=106)** | **Control group**  **(n=3,699)** | **p-value** | **PPC group**  **(n=106)** | **Control group**  **(n=106)** | **p-value** |
| Age (years), mean±SD | 53.72±17 | 53.11±14.79 | 0.7169 | 53.72±17 | 52.85±16.17 | 0.7037 |
| Sex, Male n (%) | 72(67.92) | 2314(62.56) | 0.3083 | 72(67.92) | 72(67.92) | 1.0000 |
| T2DM, n (%) | 58(54.72) | 1977(53.45) | 0.8437 | 58(54.72) | 55(51.89) | 0.7831 |
| Hypertension,  n (%) | 42(39.62) | 2022(54.66) | 0.0028 | 42(39.62) | 39(36.79) | 0.7775 |
| Hyperlipidemia, n (%) | 83(78.3) | 3231(87.35) | 0.0115 | 83(78.3) | 81(76.42) | 0.8698 |
| CVD, n (%) | 80(75.47) | 2715(73.4) | 0.7379 | 80(75.47) | 74(69.81) | 0.4413 |

AST, aspartate transaminase; PPC, polyenyl phosphatidylcholine; PSM, propensity-score matching; T2DM, type 2 diabetes mellitus; CVD, cardiovascular disease.

Table S3: Baseline characteristics before and after propensity score matching (TBiL 12 weeks)

| **Baseline variables** | **Before PSM** | | | **After PSM** | | |
| --- | --- | --- | --- | --- | --- | --- |
|  | **PPC group (n=98)** | **Control group (n=5,411)** | **p-value** | **PPC group (n=98)** | **Control group (n=98)** | **p-value** |
| Age (years), mean±SD | 53.80±16.98 | 52.92±15.35 | 0.4937 | 53.80±16.98 | 53.89±16.92 | 0.9327 |
| Sex, Male n (%) | 67(68.37) | 3446(63.69) | 0.3964 | 67(68.37) | 67(68.37) | 1.0000 |
| T2DM, n (%) | 53(54.08) | 2951(54.54) | 1.0000 | 53(54.08) | 53(54.08) | 1.0000 |
| Hypertension, n (%) | 40(40.82) | 2834(52.37) | 0.0248 | 40(40.82) | 39(39.80) | 1.0000 |
| Hyperlipidemia, n (%) | 81(82.65) | 4628(85.53) | 0.3882 | 81(82.65) | 80(81.63) | 1.0000 |
| CVD, n (%) | 42(42.86) | 3174(58.66) | 0.0019 | 42(42.86) | 42(42.86) | 1.0000 |

TBiL, total bilirubin; PPC, polyenyl phosphatidylcholine; PSM, propensity-score matching; T2DM, type 2 diabetes mellitus; CVD, cardiovascular disease.

Table S4: Baseline characteristics before and after propensity score matching (TBiL 24 weeks)

| **Baseline variables** | **Before PSM** | | | **After PSM** | | |
| --- | --- | --- | --- | --- | --- | --- |
|  | **PPC group (n=52)** | **Control group (n=6,334)** | **p-value** | **PPC group (n=52)** | **Control group (n=52)** | **p-value** |
| Age (years), mean±SD | 58.88±16.11 | 54.10±14.92 | 0.0246 | 58.88±16.11 | 58.88±15.76 | 1.0000 |
| Sex, Male n (%) | 27(51.92) | 4034(63.69) | 0.0837 | 27(51.92) | 27(51.92) | 1.0000 |
| T2DM, n (%) | 34(65.38) | 3532(55.76) | 0.2066 | 34(65.38) | 34(65.38) | 1.0000 |
| Hypertension, n (%) | 26(50.00) | 3320(52.42) | 0.7811 | 26(50.00) | 26(50.00) | 1.0000 |
| Hyperlipidemia, n (%) | 41(78.85) | 5444(85.95) | 0.1585 | 41(78.85) | 41(78.85) | 1.0000 |
| CVD, n (%) | 26(50.00) | 3756(59.30) | 0.2022 | 26(50.00) | 26(50.00) |  |

TBiL, total bilirubin; PPC, polyenyl phosphatidylcholine; PSM, propensity-score matching; T2DM, type 2 diabetes mellitus; CVD, cardiovascular disease.

Table S5: Baseline characteristics before and after propensity score matching (LDL-C 12 weeks)

| **Baseline variables** | **Before PSM** | | | **After PSM** | | |
| --- | --- | --- | --- | --- | --- | --- |
|  | **PPC group (n =71)** | **Control group (n=3,911)** | **p-value** | **PPC group (n =71)** | **Control group (n=71)** | **p-value** |
| Age (years), mean±SD | 55.28±16.94 | 51.99±14.89 | 0.6758 | 55.28±16.94 | 54.73±16.54 | 0.7946 |
| Sex, Male n (%) | 48(67.61) | 2605(66.61) | 0.9264 | 48(67.61) | 48(67.61) | 1.0000 |
| T2DM, n (%) | 34(47.89) | 2113(54.03) | 0.0012 | 34(47.89) | 31(43.66) | 0.7364 |
| Hypertension, n (%) | 34(47.89) | 2014(51.50) | 0.1908 | 34(47.89) | 33(46.48) | 1.0000 |
| CVD, n (%) | 34(47.89) | 2239(57.25) | 0.0081 | 34(47.89) | 33(46.48) | 1.0000 |

LDL-C, Low-Density Lipoprotein Cholesterol; PPC, polyenyl phosphatidylcholine; PSM, propensity-score matching; T2DM, type 2 diabetes mellitus; CVD, cardiovascular disease.

Table S6: Comparison of baseline characteristics (FIB-4 24-weeks)

| **Baseline variables** | **FIB-4 group**  **(n=42)** | **Underwent PPC monotherapy over 24 weeks (n=291)** | **p-value** |
| --- | --- | --- | --- |
| Age (years), mean±SD | 57.98±17.11 | 61.55±14.34 | 0.2031 |
| Sex, Male n (%) | 25(59.52) | 189(64.95) | 0.4955 |
| T2DM, n (%) | 26(61.9) | 183(62.89) | 1.0000 |
| Hypertension, n (%) | 23(54.76) | 164(56.36) | 0.8691 |
| Hyperlipidemia, n (%) | 33(78.57) | 220(75.6) | 0.8470 |
| CVD, n (%) | 24(57.14) | 186(63.92) | 0.3979 |

FIB-4, Fibrosis-4, age ([yr] x AST [U/L]) / ((PLT [10(9)/L]) x (ALT [U/L])(1/2));PPC, polyenyl phosphatidylcholine; T2DM, type 2 diabetes mellitus; CVD, cardiovascular diseas

Table S7: Comparison of baseline characteristics (AST)

| **Baseline variables** | **AST group (n=106)** | **Underwent PPC monotherapy over 12 weeks (n=675)** | **p-value** |
| --- | --- | --- | --- |
| Age (years), mean±SD | 53.72±17 | 58.25±15.72 | 0.0150 |
| Sex, Male n (%) | 72 (67.92) | 441(65.33) | 0.6604 |
| T2DM, n (%) | 58 (54.72) | 401(59.41) | 0.3962 |
| Hypertension, n (%) | 42 (39.62) | 351(52.00) | 0.0213 |
| Hyperlipidemia, n (%) | 83 (78.30) | 519(76.89) | 0.8046 |
| CVD, n (%) | 45(42.45) | 392(58.07) | 0.0031 |

AST, aspartate aminotransferase; PPC, polyenyl phosphatidylcholine; T2DM, type 2 diabetes mellitus; CVD, cardiovascular disease.
